# The latest FAD – Faecal antibody detection in cattle. Protocol and results from three UK beef farms naturally infected with gastrointestinal nematodes

**DOI:** 10.1017/S0031182018000902

**Published:** 2018-08-08

**Authors:** A. S. Cooke, K. A. Watt, E. R. Morgan, J. A. J. Dungait

**Affiliations:** 1 Sustainable Agriculture Sciences, Rothamsted Research, North Wyke, Okehampton, Devon EX20 2SB, UK; 2 School of Veterinary Science, University of Bristol, Langford House, Langford, North Somerset BS40 5DU, UK; 3 Institute of Evolutionary Biology, University of Edinburgh, Ashworth Laboratories, The Kings Buildings, Charlotte Auerbach Road, Edinburgh EH9 3FL, UK; 4 Institute for Global Food Security, Queen's University Belfast, University Road, Belfast BT7 1NN, UK

**Keywords:** Agriculture, antibody, diagnostics, disease, health, immunoglobulin, immunology, one-health, parasite, parasitology, veterinary

## Abstract

Antibodies at gastrointestinal mucosal membranes play a vital role in immunological protection against a range of pathogens, including helminths. Gastrointestinal health is central to efficient livestock production, and such infections cause significant losses. Fecal samples were taken from 114 cattle, across three beef farms, with matched blood samples taken from 22 of those animals. To achieve fecal antibody detection, a novel fecal supernatant was extracted. Fecal supernatant and serum samples were then analysed, using adapted enzyme-linked immunosorbent assay protocols, for levels of total immunoglobulin (Ig)A, IgG, IgM, and *Teladorsagia circumcincta*-specific IgA, IgG, IgM and IgE (in the absence of reagents for cattle-specific nematode species). Fecal nematode egg counts were conducted on all fecal samples. Assays performed successfully and showed that IgA was the predominant antibody in fecal samples, whereas IgG was predominant in serum. Total IgA in feces and serum correlated within individuals (0.581, *P* = 0.005), but other Ig types did not. Results support the hypothesis that the tested protocols are an effective method for the non-invasive assessment of cattle immunology. The method could be used as part of animal health assessments, although further work is required to interpret the relationship between results and levels of infection and immunity.

## Introduction

Infection of cattle with parasites, especially gastrointestinal nematodes (GINs), incurs important economic losses, while options for control are undermined by anthelmintic drug resistance. Targeted selective treatment (TST), whereby drugs are provided only to individuals in greatest need, has the potential to yield long-term benefits to animal health at individual, herd and national levels due to its mitigating effect on the selection of drug-resistant pathogens (van Wyk *et al.*, 2006; Charlier *et al.*, 2014). Central to TST strategies is the need for comprehensive animal health assessments, used to select individuals for treatment (Bath and van Wyk, 2009; Bentounsi *et al.*, 2012; Charlier *et al.*, 2014). This screening process can involve a range of non-specific health indicators, such as weight gain, body condition and evidence of diarrhoea, in tandem with more specific indicators of infection such as fecal egg counts (FECs). A significant drawback of FEC techniques is that egg counts are not necessarily indicative of parasite burden, or of consequent pathology or impact on health. The advancement of TST requires the development of new, high throughput diagnostics that are able to assess physiological parameters of animal health, especially in relation to GINs. Fecal antibody detection (FAD) is a candidate to join this tool kit of techniques, allowing for more detailed and comprehensive evaluations of animal health, therefore enhancing current TST strategies.

Gastrointestinal health is particularly important for efficient feed conversion within livestock production systems and general animal health. The gut wall acts as an interactive barrier between the external environment and the rest of the animal's systems, allowing for the passage of beneficial nutrients into the body. However, the gut wall is also a primary entry point and barrier for ingested pathogens that have the potential to cause extensive physiological damage and ultimately reduce nutrient utility and subsequent animal health and performance (Sykes *et al.*, 1975; Smith *et al.*, 1985; Parkins and Holmes, 1989; Poppi *et al.*, 1990; Coop and Holmes, 1996; Claerebout and Vercruysse, 2000). A key component in this defence against pathogens is the immune system and its response at mucosal membranes (Miller, 1987; Nawa *et al.*, 1994; Onah and Nawa, 2000; Nagler-Anderson, 2001; Sansonetti, 2004) including immunoglobulins (Ig), which directly combat pathogens and other foreign bodies. Each of the five Ig isotypes (IgA, IgD, IgE, IgG and IgM) has numerous subtypes that play different roles and are generally localized to specific systems or tissues.

Heightened antibody levels are often symptomatic of disease challenge and an individual's response to that; however, challenge does not necessarily relate to pathology if an animal is coping (Newkirk *et al.*, 2005; Dong *et al.*, 2008). Over the course of an infection, antibody levels vary greatly and are typically characterized by a primary and secondary response; therefore, antibody levels at a single time point may not necessarily correlate with disease burden. Antibody levels are widely measured to indicate exposure, response and tolerance of hosts to GINs and other pathogens. This is practised most commonly in the dairy industry, with testing of bulk-tank milk samples used to assess herd health and inform disease control strategies (Nielsen *et al.*, 2000; Stabel *et al.*, 2002; Sekiya *et al.*, 2013; Parker *et al.*, 2017). A limitation of antibody quantification in non-milk-yielding individuals is the necessity for an invasive sampling procedure. A second limitation is that different tissues will have a different balance of antibodies due to their source, such that milk antibody levels are not directly comparable to serum antibody levels, and neither necessarily reflect mucosal immunity. There is therefore a need for a non-invasive method of antibody quantification that is applicable to all individuals, irrespective of age, gender and other variables. Furthermore, FAD has the potential to more directly derive information regarding gastrointestinal health than equivalent diagnostics on milk or serum.

For the purposes of animal health investigation, enzyme-linked immunosorbent assays (ELISAs) are typically conducted on plasma, serum or milk samples. However, a small number of studies have utilized animal fecal samples collected in the laboratory (Wedrychowicz *et al.*, 1985) and field (Peters *et al.*, 2004; Watt *et al.*, 2015). Watt *et al*. (2015) specifically measured antibodies of *Teladorsagia circumcincta*, a GIN. While *T. circumcincta* is predominantly a parasite of sheep, there is mounting evidence for the cross-reactivity of antibodies, produced against antigens of a specific nematode, to other GIN species (Blanchard and Wescott, 1985; Molina *et al.*, 1999; Ruma *et al.*, 2016). Immunological results from fecal material are more likely to be representative of gastrointestinal mucosal membranes than those from other tissues such as plasma, due to the physiologically localized nature of immunity (Lamm, 1988; Wennerås *et al.*, 1999), therefore providing novel and complementary information about animal health.

There is the potential for FAD ELISAs to allow for quantitative assessment of the immunological status of gastrointestinal mucosal membranes as a function of general animal health, gut health or GIN challenge. Fecal material is easily and commonly collected for the purpose of FECs. The benefits of FAD and the novel information it can provide makes it a promising technique for the future veterinary, agricultural and zoological studies into animal health.

The primary objective of the research presented here was to determine the feasibility of a cattle fecal supernatant as a suitable material for quantitative detection of antibodies using ELISA. Further, to assess if fecal antibody levels are representative of those at mucosal membranes: spefically, whether IgA is the most abundant antibody in both cases. Finally, to assess whether fecal antibody levels correlate with serum antibody levels and FEC.

## Methods

### Sample collection and processing

#### Sample herds

Fecal samples were taken from cattle at three UK beef farms.

Farm #1 was at Rothamsted Research's North Wyke Farm Platform, in Devon, England. The Farm Platform has three non-organic, pasture-fed beef herds, under typical managed rotation. Each herd is similar but grazes on different pasture systems. An initial sampling on 10/11/2016 collected 45 fecal samples and the second sampling on 07/02/2016 collected 18 fecal samples, six of which were from animals sampled the first time around. Both sampling instances occurred during housing when animals were on a locally produced silage diet.

Farm #2 was a pasture-fed beef farm in Hertfordshire, England. Animals were mob-grazed, i.e. frequently moved to new pasture, with movement approximately every 3 days. Sampling occurred once, on 02/02/2017, during housing, when 30 fecal and 22 blood samples were taken from 30 individuals. The farm was organic (soil association certified) and no anthelmintic treatment had been administered during the monitored season.

Farm #3 was a pasture-fed beef farm in Angus, Scotland. Cattle were mob-grazed and moved between fields up to three times per day. Sampling occurred once, on 07/12/2017, and resulted in the collection of fecal samples from 30 animals. Animals grazed year-round with no housing. The farm was organic (soil association certified) and no anthelmintic treatment had been administered during the monitored season.

#### Blood serum

Tail venepuncture was conducted, by a trained and licensed veterinarian, from 22 individuals on farm #2, to withdraw blood for regulated disease testing; sub-samples were taken for FAD. Blood samples were only collected from animals for which matched dung samples were available, and blood and fecal samples were taken on the same day. Samples were drawn, by sterile syringe, into labelled 10 mL BD Vacutainers^®^ and rested for >30 min to allow for clotting. Samples were then centrifuged at 2500 rpm/1056 × ***g*** (Sorvall SLA-3000 rotor in a Sorvall RC-5B centrifuge) for 15 min and the supernatant serum withdrawn, using sterile pipette tips, into 1.5 mL microcentrifuge tubes (Thermo Scientific™ 3451). Samples were immediately stored at −20 °C until analysis.

#### Fecal supernatant

A dung supernatant was obtained by the dilution of fresh cattle dung with a protease inhibitor, centrifuging and withdrawal of supernatant.

Fresh dung was collected from individual animals immediately after deposition, using a clean, single-use polystyrene spoon. Dung was homogenized by stirring before collection, with care taken not to mix in foreign matter such as other dung and hay. Collected samples were transferred to sterile polystyrene screw-top containers. During sampling, the samples were stored in a cool box with ice packs, after which they were stored at −20 °C until being processed.

In order to create the supernatant, dung samples were allowed to defrost at room temperature (approximately 3 h). Defrosted samples were thoroughly mixed using sterile inoculating needles (Camlab, UK). Between 2 and 4 g of dung was then transferred to a sterile beaker and mixed with a protease inhibitor (cOmplete, EDTA-free Protease Inhibitor Cocktail, Roche, Basel, Switzerland) at a recorded ratio of between 1:1 and 1:2 (w/v). The resulting mixture was homogenized using sterile inoculating needles and then transferred to sterile 10 mL centrifuge tubes (Oak Ridge High-Speed PPCO, Nalgene, USA) and rested on ice for >10 min, until centrifuging. Samples were centrifuged at 3–6 °C and 8400 rpm/12 000 × ***g*** (Sorvall SLA-3000 rotor in a Sorvall RC-5B centrifuge, ThermoFisher Scientific, Waltham, Massachusetts, USA) for 5 min. The supernatant was then pipetted, using sterile pipette tips, into 1.5 mL microcentrifuge tubes (Thermo Scientific, USA). Samples were immediately stored at −20 °C until analysis.

Three negative control protease inhibitor blanks for the supernatant diluent were created, comprising of 100% protease inhibitor cocktail. Each blank came from a different batch of inhibitor cocktail and was prepared separately.

### Assay protocol

Seven, bovine-specific ELISAs were conducted. Total IgA, IgG and IgM ELISAs were conducted using bovine-specific commercial components (Bethyl Laboratories Inc., Montgomery, Texas, USA) and a reference serum, per the manufacturer's protocol. A further three ELISAs were conducted using *T. circumcincta* antigen, measuring the responses of bovine-specific IgA, IgG, IgM and IgE to the antigen. No commercial bovine-specific IgE components were available, so a fourth assay was completed using a sheep IgE ELISA. These latter ELISAs were conducted using the same protocol as for the commercial ELISAs with the alteration that the commercial capture antibody was replaced with a *T. circumcincta* antigen, as per (Watt *et al.*, 2015). No IgD antibodies were available for inclusion.

Each ELISA was conducted on all 114 fecal supernatant samples and 22 serum samples. Each of the total Ig plates contained a 10-point dilution series of reference material and two or more blanks of TBST (Tris-buffered saline with Tween20 at 0.05%), representing the sample diluent. Three protease inhibitor blanks were also included in each assay. The *T. circumcincta* assays do not have a reference serum available, so had a known positive sample included twice, which showed that the assay worked on that day. The positive control was serum from sheep that had been trickle infected with *T. circumcincta* and had confirmed antigens against L3 *T. circumcincta*, as per (Watt *et al.*, 2015).

#### Sample dilution

Supernatant and sera had to be diluted to ensure that optical densities (ODs) were within the detection limits set by the sigmoidal curve. Samples were serial diluted and six concentrations (later narrowed down to three) taken forward for use in assays. For each assay and material, one dilution was chosen across all samples as the one to derive results from (see Supplementary material).

#### Laboratory procedure

Ninety-six-well plates (Nun-Immuno MicroWell MaxiSorp, ThermoFisher Scientific) were coated with 50 *µ*L of the matched rabbit anti-bovine antibody, diluted to 2 *µ*g mL^−1^ in 0.06 m carbonate buffer. For the *T. circumcincta* assays, the coat was *T. circumcincta* L3 somatic antigen at 2 *µ*g mL^−1^ in 0.06 m carbonate buffer. Plates were then covered in a cling film, and stored for 1–3 days at 4 °C prior to use. Plates were removed from the refrigerator and washed 3× in TBST. Meanwhile, samples were defrosted at room temperature (approximately 1 h) and then serial diluted in 2 mL deep-well plates. Fifty microlitres of the appropriate sample dilutions were pipetted into the relative wells on the plate. TBST and protease inhibitor-negative controls were then added. For total antibody assays, the serial dilutions of reference serum were added, acting as a positive control, but also providing concentration curves for later interpolation. For *T. circumcincta* assays, a known positive sheep sample was used as a plate-positive control on the four assays. Plates were then covered in a cling film and incubated for 1 h at 37.5 °C.

Plates were removed from the incubator and washed 5× in TBST. Fifty microlitres of the appropriate rabbit anti-bovine HRP-conjugated antibody was added to each plate (excluding for the *T. circumcincta* IgE assay). No direct HRP-conjugated antibody was available for the *T. circumcincta* IgE assay and instead 50 *µ*L of mouse anti-ovine IgE (monoclonal IgG1) at 10 *µ*L mL^−1^ with TBST was added. *Teladorsagia circumcincta* IgE plates were then incubated for 1 h at 37.5 °C, washed 5× with TBST and then 50 *µ*L of goat anti-mouse IgG1-HRP detection, at 0.125 *µ*g mL^−1^ with TBST, was added. All plates were then covered in a cling film and incubated for 1 h at 37.5 °C.

After incubation, plates were washed 5× in TBST. One hundred microlitres of TMB substrate (KPL, Gaithersburg, Maryland, USA, SureBlue™ TMB Microwell Peroxidase Substrate – single component) was added to each well, plates were then incubated, in darkness, for 5 min at 37.5 °C. Plates were removed from the incubator and 100 *µ*L of the stop solution, 1.0 m HCl, was added to each well (the addition of HCL inhibits enzyme activity and changes the wells from blue to yellow). Plates were immediately read by a plate reader at 450 nm, providing the OD for each well.

#### Interpolation and adjustment

For each assay quantifying abundances of a total antibody class, the 10-point dilution series was graphed as a sigmoidal curve of OD and antibody concentration. Sample ODs were interpolated onto this curve to generate an antibody concentration for each sample. These concentrations were then adjusted to account for two instances of *in vitro* sample dilution, which occurred initially when fecal supernatants were formed and again during serial dilutions of supernatants. This generated the final concentration of antibody in each fecal sample.

Due to the lack of reference material available for *T. circumcincta-*specific antibody assays, it was not possible to interpolate the results to generate an exact concentration. Instead, a relative scale was created, using the positive control, to allow for simple comparison of samples relative to one another. The value given to each sample was derived from equation (1). As per total antibody class assays, results were then adjusted to account for *in vitro* dilution. In the event that negative values were obtained (i.e. if sample OD was less than TBST OD), values were converted to zero.
1
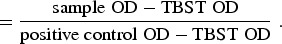


Equation (1) – Formula used to generate a relative and arbitrary scale for *T. circumcincta* antibody levels.

#### Validation

Reference material was essential to confirm the validity of assays and to calculate antibody levels. Total IgA, IgG and IgM reference material was present on each plate of their matched assay. Reference material stock concentrations for total IgA, IgG and IgM were: 0.11, 24 and 1.8 *µ*g *µ*L^−1^, respectively. Twenty-six dilutions of reference materials were formed using halving serial dilutions. The initial dilution wash 80 *µ*L of reference material with 920 *µ*L of TBST. Seven hundred microlitres of that solution was then withdrawn and added to 700 *µ*L of TBST and the process repeated to form a series of up to 26 dilutions, of which 10 were chosen for each assay (see Supplementary material). Chosen dilutions were based on the past experience of similar assays, which were then tested to ensure suitability, by visually assessing if they produced sigmoidal curves. Before experimental assays were conducted, plates were run with the specified dilutions of reference materials to confirm that the generated curves were suitable and within the detection limits of the assay and plate reader, each assay was repeated five times and plates included two blanks of TBST. As no *T. circumcincta* antibody reference material was available, and therefore could not be quantified, results were measured in relation to other samples. However, the *T. circumcincta-*positive controls were available and used to confirm that the assay worked.

### Fecal egg counts

FECs were conducted on all fecal samples used in the ELISA assays. In addition, each farm had FECs conducted in the grazing season leading up to sampling, with FECs conducted on 10 randomly collected samples on each of the four to seven sampling visits per farm.

FECs were completed in duplicate, using mini-Flotac and fill-Flotac devices (University of Naples Federico II, Naples, Italy) (Cringoli *et al.*, 2010; Bosco *et al.*, 2014), in accordance with manufacturer methods (5.0 g of faces in 45 mL of flotation solution), giving an analytic sensitivity of five eggs per gram (epg). A flotation solution of 1.34 g mL^−1^ zinc sulphate in deionized water was used. Total eggs counted across both wells of the mini-flotac plate were multiplied by five to determine epg.

### Statistical analysis of antibody results

#### Validations

Assay validity was confirmed using reference material results. For total IgA, IgG and IgM assays, ODs from the 10-point dilution curves were plotted and assays considered valid if sigmoidal curves were produced by the data plots. For *T. circumcincta* assays (for which no reference material was available), the assay was considered valid if the positive controls were significantly higher than TBST blanks, as determined by a two-sample *t*-test.

For fecal supernatants to be validated as a suitable medium for Ig assays, ODs of fecal supernatants, for at least one antibody type, must be higher than that of blanks. This was determined through two-sample *t*-tests individually comparing fecal supernatant antibody levels to those of TBST and fecal supernatant-negative controls.

#### Antibody levels

One-way analyses of variance (ANOVAs) with Tukey tests were used to determine if antibody concentrations differed between fecal and serum samples and if concentrations of different antibodies differed within samples. This was implemented for both the total antibody assay dataset and for the *T. circumcincta* dataset independently. Initial tests were conducted on both serum and fecal antibody levels combined, to determine differences in antibody concentrations and levels between the two materials.

Fecal and serum data were then split and the tests conducted to identify differences in the abundance of the different antibody types within those two datasets. Due to a large number of negative samples, zero value results were removed from *T. circumcincta* datasets and ANOVAs repeated.

For both the fecal supernatant and serum datasets, Pearson's correlations were performed on all antibody pairings to determine the correlations between antibody classes and subtypes. Further correlations were then conducted to compare the levels of the same antibodies between blood and fecal samples taken from the same animal on the same day. Bonferroni adjustments were made to account for multiple comparisons. Further correlations were also performed on 21 paired serum and fecal supernatants, derived from the same animal, on the same day. A final set of correlations were performed to determine if any antibody types correlated with FEC results (total nematode epg).

## Results

### Validation of protocol

#### Assay validation

**Fig. 1.**
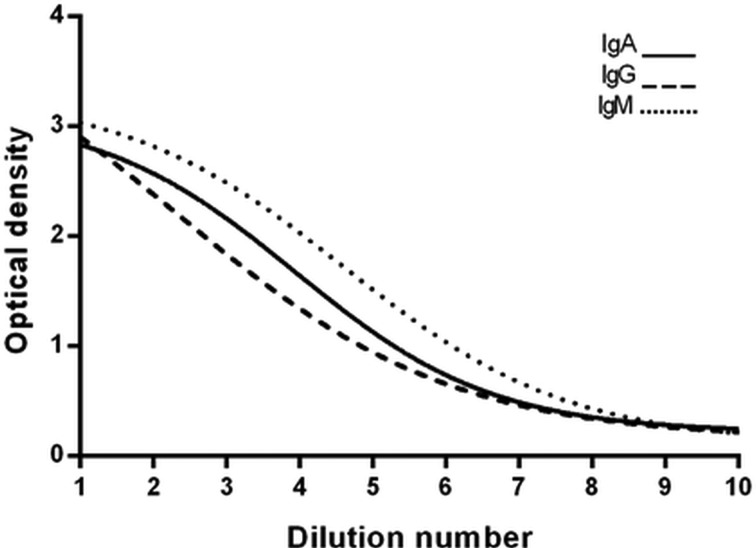
Sigmoidal curves generated from 10-point dilution series of reference material for IgA, IgG and IgM assays (total and subtype).

Ten-point dilution series for each total IgA, IgG and IgM all produced sigmoidal curves (Fig. 1).

**Fig. 2.**
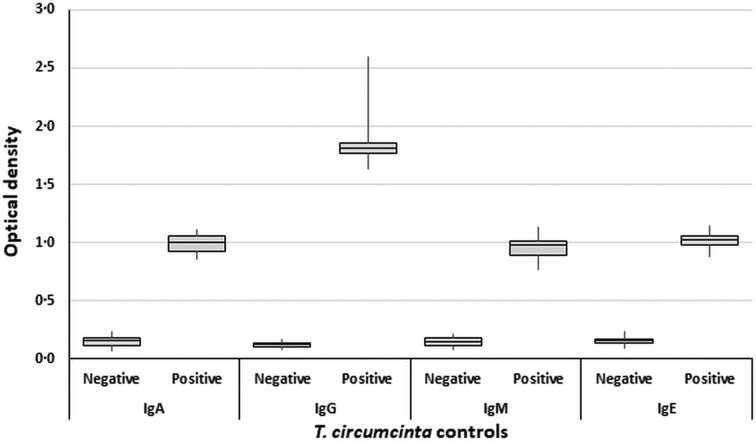
Boxplots comparing negative and positive controls for all *Teladorsagia circumcincta* assays, for the purpose of validating the ELISA.

Positive controls for the *T. circumcincta* assays yielded consistent and significantly higher ODs than the negative controls (Fig. 2). These differences were confirmed by two-sample *t*-tests for each *T. circumcincta* antibody, IgA (*T* = 25.29, *P* < 0.0005), IgG (*T* = 16.44, *P* < 0.0005), IgM (*T* = 17.79, *P* < 0.0005) and IgE (*T* = 35.39, *P* < 0.0005).

#### Fecal supernatant validation

**Fig. 3.**
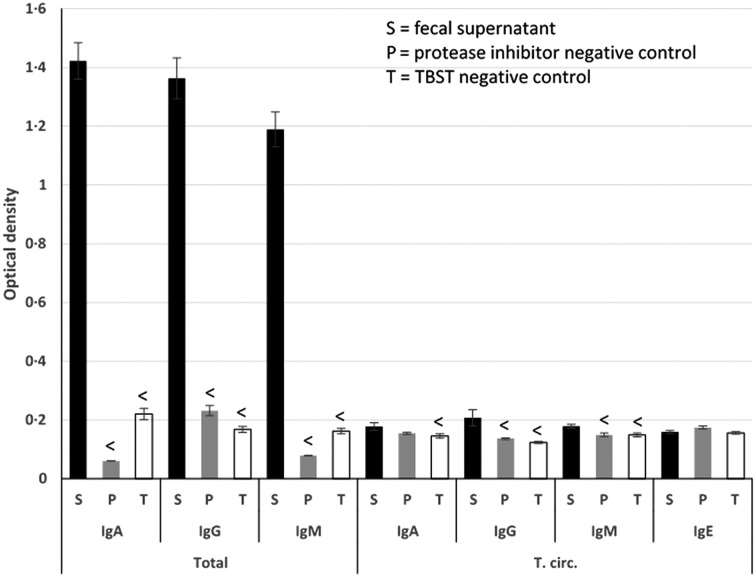
Unadjusted, ‘raw’ optical densities for fecal supernatant, protease inhibitor-negative controls and TBST-negative controls, across all assays. Less than symbols (<) above control columns signify that their ODs are statistically significantly less than the sample ODs for the same antibody, as determined by a two-sample *t*-test.

Fecal supernatant OD values were generally greater than those of TBST and protease inhibitor-negative controls (Fig. 3). The exceptions were *T. circumcincta* IgA, which was not significantly higher than its protease inhibitor control, and *T. circumcincta* IgE, which was not significantly higher than either of its blanks.

### Antibody levels

IgA was the most abundant antibody isotype in fecal samples, while IgG was the most abundant antibody isotype in serum samples.

**Fig. 4.**
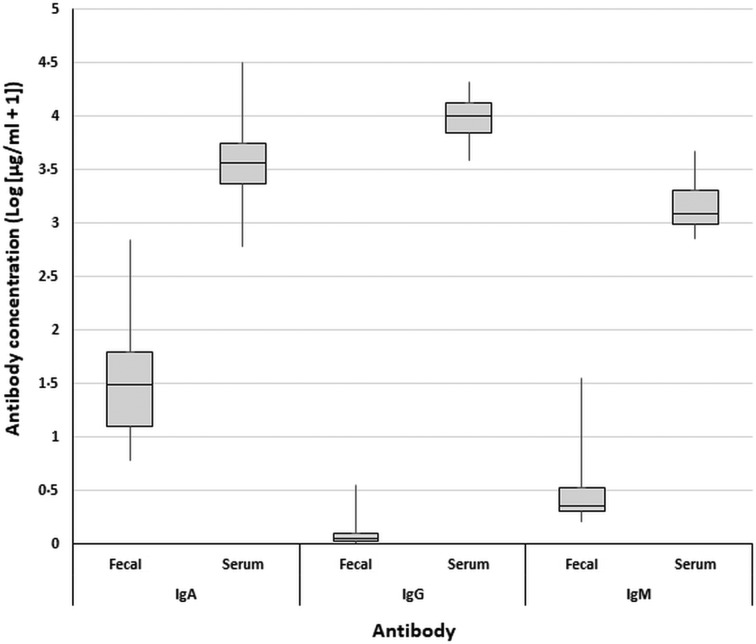
Boxplots of total antibody concentrations [log (*μ*g/mL +)] measured across all cattle fecal and serum samples.

Total antibody concentrations of positive samples varied greatly (Fig. 4). A one-way ANOVA, with a *post hoc* Tukey test, across all total antibody datasets, found that serum antibody concentrations were significantly higher than fecal antibody concentrations (*F* = 162.21, *P* < 0.0005). A second one-way ANOVA and Tukey test, comparing just fecal antibody concentrations, found fecal IgA concentrations to be significantly greater than serum IgG and IgM, which were not significantly different to one another according to the Tukey test (*F* = 50.60, *P* < 0.0005). A final one-way ANOVA and Tukey test, solely comparing serum antibody concentrations, found that serum IgG concentrations were significantly greater than serum IgA and that both were significantly greater than serum IgM (*F* = 18.97, *P* < 0.0005).

**Fig. 5.**
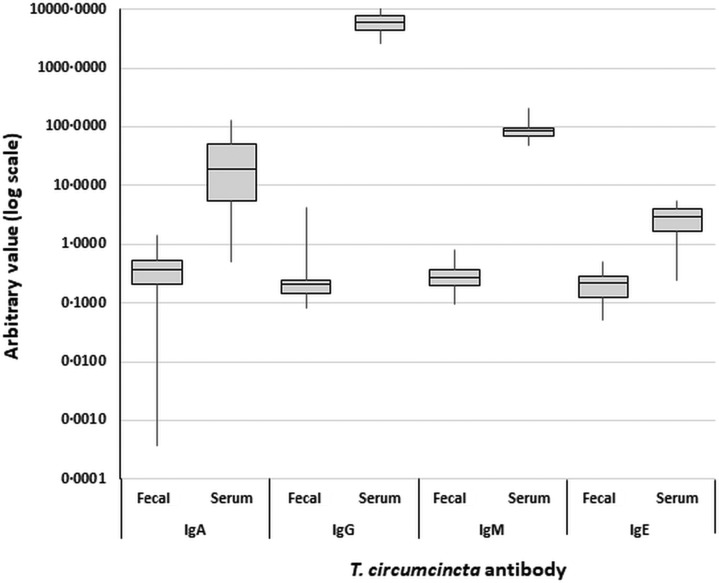
Boxplots of *Teladorsagia circumcincta*-specific antibody levels (arbitrary units) measured across all fecal and serum samples. *Y*-axis is a log scale.

The variation in *T. circumcincta* antibody levels was much less pronounced than for total antibody concentrations. A one-way ANOVA with a *post hoc* Tukey test found that *T. circumcincta* IgG levels were significantly greater than all other *T. circumcincta* antibodies in the combined dataset of feces and serum, all of which were not significantly different to one another (*F* = 548.06, *P* < 0.0005). These trends were still apparent when the fecal and serum datasets were isolated and analysed independently (*F* = 2.11, *P* = 0.098 and *F* = 152.46, *P* < 0.0005, respectively). However, when only positive samples were included in the analysis, an ANOVA and Tukey test on fecal samples found *T. circumcincta* IgA to be significantly greater than the other antibodies, which were statistically not different to one another (*F* = 4.00, *P* = 0.008) (Fig. 5).

### Fecal nematode egg counts

On farm #1, 29% of animals had GIN eggs in their feces; among these animals, the mean epg was 17 (s.e. 7.7). On farm #2, 17% of animals were positive, with a mean epg of 139 (s.e. 82.6). Farm #3 had 27% of animals recorded as positive, of which the mean epg was nine (s.e. 1.8).

### Correlations

#### Antibody correlations

The majority (14/21) fecal antibody correlations were found to be significant (Table 1). Of the seven non-significant correlations, five were for pairings that included *T. circumcincta* IgE.

**Table 1.** Pearson's correlation results for different antibodies measured within fecal samples

	IgA	IgG	IgM	*T.* IgA	*T.* IgG	*T.* IgM	*T.* IgE	
IgA		0.562*	0.700*	0.300*	0.651*	0.514*	−0.050	Correlations
IgG	<0.0005*		0.453*	0.161	0.345*	0.238	−0.203	
IgM	<0.0005*	>0.0005*		0.485*	0.640*	0.530*	−0.180	
*T.* IgA	0.001*	0.085	<0.0005*		0.781*	0.622*	0.079	
*T.* IgG	<0.0005*	<0.0005*	<0.0005*	<0.0005*		0.654*	0.011	
*T.* IgM	<0.0005*	0.007	<0.0005*	<0.0005*	<0.0005*		0.364*	
*T.* IgE	0.593	0.028	0.053	0.402	0.905	<0.0005*		
	*P* values							

The top right half of the chart shows the correlation statistics and the bottom left half shows the *P* value. Results with an asterisk (*) are statistically significant at an adjusted critical *P* value of 0.0024. ‘*T.*’ refers to *Teladorsagia circumcincta*. *n* = 116.

Of the 21 correlations conducted, only one serum antibody pairing (IgM *vs T. circumcincta* IgM) correlated significantly (Table 2).

**Table 2.** Pearson's correlation results for different antibodies measured within blood samples

	IgA	IgG	IgM	*T.* IgA	*T.* IgG	*T.* IgM	*T.* IgE	
IgA		0.487	0.418	0.161	−0.138	0.103	0.359	Correlations
IgG	0.021		0.500	0.530	0.352	0.324	0.141	
IgM	0.053	0.018		0.439	0.268	0.669*	0.445	
*T.* IgA	0.473	0.011	0.041		0.235	0.370	0.155	
*T.* IgG	0.541	0.108	0.227	0.291		0.545	−0.110	
*T.* IgM	0.650	0.141	0.001*	0.090	0.009		0.014	
*T.* IgE	0.101	0.131	0.038	0.490	0.625	0.952		
	*P* values							

The top right half of the chart shows the correlation statistics and the bottom left half shows the *P* value. Results with an asterisk (*) are statistically significant at an adjusted critical *P* value of 0.0024. ‘*T.*’ refers to *Teladorsagia circumcincta*. *n* = 22.

When comparing levels of the same antibody taken from fecal and serum samples of the same individuals on the same day, the only significant correlation found was with IgA concentrations (Table 3).

**Table 3.** Pearson's correlations comparing levels of the same antibodies from both fecal and serum samples taken from the same individual on the same day

	IgA	IgG	IgM	*T.* IgA	*T.* IgG	*T.* IgM	*T.* IgE
Correl.	0.581*	0.511	0.010	−0.028	−0.207	0.173	0.103
*P* value	0.005*	0.105	0.966	0.900	0.354	0.442	0.648

Results with an asterisks (*) are statistically significant at an adjusted *P* value of 0.0071. ‘*T.*’ refers to *Teladorsagia circumcincta*. *n* = 22.

#### Antibody *vs* FEC

FECs correlated negatively with all antibody types; however, correlations were all <0.1 and non-significant.

## Discussion

### Assay validity

The experiment achieved its primary objective, to quantify antibody levels in cattle feces, and is therefore considered a valid protocol. Results from reference materials and controls provided sufficient evidence that the commercial ELISA products worked effectively, providing a stable foundation from which to assess the validity of the protocol. Positive controls for the *T. circumcincta* assays also provided evidence that they too worked effectively.

The greater mean ODs observed from fecal supernatants, compared with the blanks of TBST or protease inhibitor, support the validity of a fecal supernatant as a suitable material for ELISAs. This highlights the potential for fecal material to be used in the immunological assessment of animal health, particularly cattle and other ruminants.

### Interpretation

Across fecal samples, levels of IgA, both total and *T. circumcincta* specific, were significantly higher than those of all other antibodies. This result is consistent with the literature, that IgA is by far the most abundant antibody at mucosal membranes (Hughes *et al.*, 1981; Lamm, 1988; Macpherson *et al.*, 2008). This finding supports that fecal antibody levels are indicative of mucosal membrane antibody levels, as seen in humans (Crabbé and Heremans, 1968; Tomasi, 1970; Baklien and Brandtzaeg, 1975; Bjerke *et al.*, 1986). To confirm this, post-mortem intestinal washes could be utilized to recover mucosal antibodies and other biomarkers (Negrão-Corrêa *et al.*, 1996) for comparison with those found in feces from the same individual. During gut transit, organic material, which later ends up in feces, might accumulate biomarkers from mucosal membranes, making feces a rich resource for the assessment of gut health. The most abundant antibody in serum was IgG, which is also expected given the literature (Fahey and McKelvey, 1965; Hughes *et al.*, 1981). This provides additional reassurance that the various assays accurately and proportionally represent antibody levels in the relevant tissue/material. Similar relative antibody abundances in feces were also observed in sheep by Watt *et al.* (2015).

Only 15% of fecal samples were returned as positive after FEC, providing an inadequate amount of positive data to determine with any certainty, if a correlation exists between nematode egg counts and fecal antibody levels. The negative correlations observed (although non-significant) are consistent with the observations by Watt *et al*. (2015). The lower fecal antibody levels and lack of correlation with FECs may stem from hypobiosis as samples were taken during late autumn and early winter (Capitini *et al.*, 1990). Moreover, observed FECs were rather low. A longitudinal study, tracking seasonal fecal antibody trajectories would clarify this and potentially provide a more suitable FEC dataset for analysis.

Assays for *T. circumcincta*-specific antibodies were only able to provide relative and arbitrary results due to there being no available reference material. The total antibodies standard curves could not be used for interpolation of *T. circumcincta* antibodies as the relative avidities of both capture antibodies is unknown. To achieve quantitative concentrations, a reference sample with a known concentration of the relative *T. circumcincta* antibodies would need to be created. This would require the artificial infection of a host animal (likely sheep), with *T. circumcincta*, followed by slaughter and measurement of antibody concentrations in the blood, which was not a viable option in the current work.

### Application

The absence of a correlation between blood and fecal antibody levels shows that the method is not a replacement or proxy for measurements of systemic antibody levels. However, results support the utility of FAD to derive specific information about animal health that cannot easily be obtained otherwise. This information may prove to be of greater use and relevance for the assessment of GIN derived, and other, gut damage, than circulating serum antibodies. Similar recent advances have seen the development and adoption of salivary antibody tests, for the study of GIN in sheep (Shaw *et al.*, 2012). The Carla Saliva Test detects Carla antibodies (Harrison *et al.*, 2003) in sheep saliva; however, these antibodies are also present in gastrointestinal mucus, meaning that FAD may be a suitable approach for measuring Carla antibodies. The primary disadvantage of a salivary test, compared with FAD, is the necessity to perform an invasive procedure on a restrained animal. Research and development of FAD methodologies and associated technologies, using advancements on salivary antibody tests as a template, has the potential to create a highly practical and informative diagnostic method.

It is evident that FAD has the ability to quantify symptomatic and important aspects of animal immunology; however, there is limited understanding about what precisely fecal antibody levels indicate, especially in relation to pathogen-driven pathology. Given the multi-functional role of antibodies, FADs may be best used as a general marker of animal gut health and disease challenge, particularly from gastrointestinal pathogens such as GINs, and applied as part of a TST strategy. Larger scale and more longitudinal studies are necessary to further understand how FAD could best be utilized.

Detection of molecules within feces need not be restricted to antibodies, and there are a range of biomarkers to which the outlined protocols might be adapted. Two prime candidate molecules are the inflammatory markers: lactoferrin and calprotectin, which are routinely quantified within human medicine, for the diagnosis of bowel diseases (Røseth *et al.*, 1999; Lundberg *et al.*, 2005; Gisbert *et al.*, 2009; Lamb and Mansfield, 2011). Furthermore, gut inflammation can be symptomatic of GIN damage. Lactoferrin is monitored in milk as part of quality and safety assurance, therefore bovine assays are commercially available. Pepsinogen and gastrin ELISAs can be used as veterinary immunodiagnostic tools for GIN infections and are therefore also strong candidates for fecal detection, given their established utility as immuno-markers (Berghen *et al.*, 1993; Charlier *et al.*, 2011).

The outlined protocols produce a large amount of fecal supernatant, providing enough for multiple assays. Once protocols have been developed, throughput can be extremely high; within this study, for example, sixteen 96-well plates could be completed manually within one day. This number could be increased, for example, with automated pipetting machines. This brings about the possibility of fecal supernatants being used to provide a wealth of immunological data, paired with other measures of animal health, as part of a comprehensive and longitudinal animal health assessment, driving highly targeted individual interventions to support efficient and sustainable disease control.

In conclusion, the results presented advance the potential of animal feces as a resource for veterinary diagnostics. Consistent positive Ig levels, above background levels, combined with the range and distribution of results, support the methodology as a valid immunological tool. Results indicated that fecal antibody levels are representative of gastrointestinal immunology, due to the similarity in antibody profiles of fecal material compared with those observed at mucosal membrane surfaces, with IgA being the most abundant antibody (Lamm, 1988; Mazanec *et al.*, 1993; Macpherson *et al.*, 2008). This is also in-keeping with the passage and processing of material through the gut and into feces. Therefore, FAD has the potential to provide novel and unique information about gastrointestinal health and immunology.

FAD is a new, but promising, capability to assess immunological aspects of ruminant gut health in a timely and cost-effective manner. The method is highly ethical as it is non-invasive, which brings the additional benefit of not requiring trained veterinarians or licensing under animal protection legislation. For more comprehensive interpretation of fecal antibody levels, further work needs to be performed to determine the drivers of fecal antibody concentrations, most notably the role of pathogens. Successful FAD protocols within this study and by Watt *et al*. (2015) suggest that FAD might be more widely applicable to other mammals, particularly ruminants. Further advancements in the detection of fecal immuno-markers could, in the future, become part of a comprehensive tool kit for the assessment of animal health and development of disease prevention strategies.

## Supplementary material

For supplementary material accompanying this paper visit https://doi.org/10.1017/S0031182018000902. click here to view supplementary material
