# Supplementary material for: The latest FAD – Faecal antibody detection in cattle. Protocol and results from three UK beef farms naturally infected with gastrointestinal nematodes
Source: Parasitology. 2018 Aug 8;146(1):89–96. doi: 10.1017/S0031182018000902 (PMC6230468; doi:10.1017/S0031182018000902)
Supplement: Supplementary file 1 [file S0031182018000902sup001.docx]

# Supplementary material

## Bovine Reference serum material dilutions used in Total assays only

Table 1 - Serial dilutions for reference material. Asterisks (*) represent the dilutions which were used on plates as reference dilution series.

| **Dilution no.** | **Dilution x1** | **Antibody concentration (µg/ml)** | | |
| --- | --- | --- | --- | --- |
|  |  | **IgA** | **IgG** | **IgM** |
| **0** | **1 (stock)** | 110 | 2400 | 1800 |
| **1** | **2** | 8.8 | 192 | 144 |
| **2** | **4** | 4.4 | 96 | 72 |
| **3** | **8** | 2.2 | 48 | 36 |
| **4** | **16** | 1.1 | 24 | 18 |
| **5** | **32** | 0.55* | 12 | 9 |
| **6** | **64** | 0.275* | 6 | 4.5 |
| **7** | **128** | 0.1375* | 3 | 2.25 |
| **8** | **256** | 0.06875* | 1.5 | 1.125 |
| **9** | **512** | 0.034375* | 0.75 | 0.5625 |
| **10** | **1024** | 0.0171875* | 0.375 | 0.28125* |
| **11** | **2048** | 0.00859375* | 0.1875 | 0.140625* |
| **12** | **4096** | 0.00429688* | 0.09375 | 0.070313* |
| **13** | **8192** | 0.00214844* | 0.046875 | 0.035156* |
| **14** | **16384** | 0.00107422* | 0.0234375 | 0.017578* |
| **15** | **32768** | 0.00053711 | 0.01171875 | 0.008789* |
| **16** | **65536** | 0.00026855 | 0.00585938 | 0.004395* |
| **17** | **131072** | 0.00013428 | 0.00292969* | 0.002197* |
| **18** | **262144** | 6.7139E-05 | 0.00146484* | 0.001099 |
| **19** | **524288** | 3.3569E-05 | 0.00073242* | 0.000549 |
| **20** | **1048576** | 1.6785E-05 | 0.00036621* | 0.000275 |
| **21** | **2097152** | 8.3923E-06 | 0.00018311* | 0.000137 |
| **22** | **4194304** | 4.1962E-06 | 9.1553E-05* | 6.87E-05 |
| **23** | **8388608** | 2.0981E-06 | 4.5776E-05* | 3.43E-05 |
| **24** | **16777216** | 1.049E-06 | 2.2888E-05* | 1.72E-05 |
| **25** | **33554432** | 5.2452E-07 | 1.1444E-05* | 8.58E-06 |
| **26** | **67108864** | 2.6226E-07 | 5.722E-06* | 4.29E-06 |

## Sample dilutions

Table 2 - Serial dilutions used in assays for each antibody and each sample. Asterisks (*) represent the dilutions taken forward for interpolation and subsequent statistical analysis.

|  |  | Faecal supernatant dilution x1 | | | | | | Serum dilutions x100 | | | | | |
| --- | --- | --- | --- | --- | --- | --- | --- | --- | --- | --- | --- | --- | --- |
| Total | IgA | 4 | 8* | 16 | 32 | 65 | 128 | 32 | 64* | 128 | 256 | 512 | 1024 |
|  | IgG | 4 | 8* | 16 | 32 | 65 | 128 | 8192 | 16384* | 32768 | 65536 | 131072 | 262144 |
|  | IgM | 4 | 8* | 16 | 32 | 65 | 128 | 64 | 128 | 256* | 512 | 1024 | 2048 |
| *T. circumcincta* | IgA | 1 | 2* | 4 | 8 | 16 | 32 | 0.5 | 100 | 200* | 400 | 800 | 1600 |
|  | IgG | 1* | 2 | 4 | 8 | 16 | 32 | 32 | 64* | 128 | 256 | 512 | 1024 |
|  | IgM | 1* | 2 | 4 | 8 | 16 | 32 | 0.5 | 100* | 200 | 400 | 800 | 1600 |
|  | IgE | 1* | 2 | 4 | 8 | 16 | 32 | 0.5* | 100 | 200 | 400 | 800 | 1600 |

## Primary ELISA reagent list

A list of commercially available products used for the ELISA assays. Non-specialised reagents, such as hydrochloric acid and TBST, are excluded from this list.

**Rabbit anti-Bovine IgA Antibody**

Bethyl Laboratories, Inc.

Catalogue number: A10-108

**Rabbit anti-Bovine IgA Antibody HRP Conjugated**

Bethyl Laboratories, Inc.

Catalogue number: A10-108P

**Rabbit anti-Bovine IgG-heavy and light chain Antibody**

Bethyl Laboratories, Inc.

Catalogue number: A10-102

**Rabbit anti-Bovine IgG-heaving and light chain Antibody HRP conjugated**

Bethyl Laboratories, Inc.

Catalogue number: A10-102P

**Rabbit anti-Bovine IgM Antibody Affinity Purified**

Bethyl Laboratories, Inc.

Catalogue number: A10-100A

**Rabbit anti-Bovine IgM Antibody HRP Conjugated**

Bethyl Laboratories, Inc.

Catalogue number: A10-100P

**Goat anti-mouse IgG1-HRP detection antibody**

AbD Serotec

Catalogue number: STAR132P

**Bovine Reference Serum**

Bethyl Laboratories, Inc.

Catalogue number: RS10-103

**SureBlue™ TMB Microwell Peroxidase Substrate Kit**

KPL

Catalogue number: 52-00-02
